# Associations among mental health promotion, sleep concerns, fatigue, and stress in clinical nurses: network and latent profile analysis

**DOI:** 10.3389/fpubh.2026.1810766

**Published:** 2026-07-16

**Authors:** Min Dong, Lingjie Xu, Yongxia Chen, Yue Yang, Qi Xie, Mingyu Li, Rongrong Ma, Yanru Li

**Affiliations:** First Affiliated Hospital of Bengbu Medical University, Bengbu, China

**Keywords:** fatigue, latent profile analysis, mental health promotion, network analysis, nurses, sleep concerns, stress

## Abstract

**Background:**

The demanding nature of modern healthcare systems is associated with substantial fatigue and stress among clinical nurses; however, the joint associations of mental health promotion and sleep concerns with fatigue and stress among nurses remain to be elucidated.

**Methods:**

A multicenter cross-sectional survey was conducted among clinical nurses from three tertiary hospitals in Anhui Province, China. Mental health promotion, sleep concerns, fatigue, and perceived stress were assessed using the Mental Health Promotion Scale, the Anxiety and Preoccupation about Sleep Questionnaire, the Multidimensional Fatigue Inventory, and the Chinese version of the Perceived Stress Scale, respectively. The complex relationships among these variables, and characterizations of different subgroups, were studied using network analysis and latent profile analysis.

**Results:**

All 352 distributed questionnaires were returned, of which 338 valid questionnaires were included in the analysis. Network analysis showed that certain dimensions of mental health promotion (values, personal development, and friendship) were negatively correlated with both fatigue and stress, while sleep concerns, particularly sleep-related preoccupation, were positively correlated with fatigue and stress. Within the network, values showed the highest strength centrality. Physical fatigue, tension, and sleep-related preoccupation were key bridging nodes connecting different construct communities. Latent profile analysis identified three statistically derived fatigue/stress profiles: low fatigue/low stress (*n* = 33), moderate fatigue/high stress (*n* = 241), and high fatigue/high stress (*n* = 64).

**Conclusion:**

Mental health promotion showed a negative association with fatigue and stress among clinical nurses, with values representing a core dimension of mental health resources. Furthermore, sleep-related preoccupation was observed as a bridging node between psychological and physical dimensions. These findings identify occupational values, physical fatigue, tension, and sleep-related preoccupation as candidate constructs for evaluation in future longitudinal and intervention studies.

## 1 Introduction

The workload within modern healthcare systems is increasingly demanding, resulting in an ever-growing toll on the physical and psychological health of clinical nurses. Frequent night and rotating shifts disrupt normal circadian rhythms (1), with recent data showing that up to 66.4% of nurses suffer from poor sleep quality (2). Additionally, within the context of the job, nurses tend to expend a massive amount of psychological energy providing emotional support to patients and their families (3). Together, these occupational demands are associated with fatigue and stress (4), which are closely related to burnout, anxiety, and depression (5). Ultimately, these working conditions have been associated with poorer physical and mental health among nurses and with patient-safety concerns (6).

Current mental health promotion programs targeting nurse fatigue and stress have integrated psychological, organizational, and behavioral factors in an effort to enhance resilience and alleviate work-related stress (7). These programs are based on the Job Demand–Resource (JD-R) framework (8), which conceptualizes psychological resources (i.e., reinforced professional values, family/social support, and personal growth opportunities) as key elements that may help nurses cope with job demands and manage occupational burden. Guided by the JD-R model, the current study conceptualizes mental health promotion as a personal resource, sleep concerns as a sleep-related cognitive burden, and fatigue and perceived stress as strain outcomes, and explores how these factors are associated in nurses. Healthcare workers actively construct their professional experience through the lens of their personal resources and social support systems, which highlights the potential role of the organizational environment in occupational stress. In the clinical setting, nurses are not passive recipients of job demands, but active participants whose psychological resources may be associated with their cognitive assessment and coping behaviors in response to work stressors (9). Persistent sleep-related anxiety, especially about poor sleep quality and shift work-related circadian rhythm disruptions, has been associated with lower psychological capital (10), poorer professional performance (11), and a higher risk of clinical errors. Neurocognitive studies have proposed anticipatory anxiety and attention deficits as possible mechanisms linking sleep-related anxiety with fatigue and stress (12). Exploring how mental health promotion and sleep concerns are jointly associated with fatigue and stress in nurses has theoretical and occupational-health relevance. In this study, the JD-R framework was used as a theory-informed interpretive framework to position mental health promotion as a personal resource, sleep concerns as a sleep-related cognitive burden, and fatigue and perceived stress as strain-related outcomes rather than as a fully tested explanatory or causal model.

While appropriate psychological resources are associated with better physical and mental health among nurses, chronic workplace stress may offset these favorable associations. For example, Chen et al. (13) identified several nurse subgroups with different stress adaptation patterns through the application of latent trait analysis, and stress-induced burnout was associated with poorer mental state and cognitive abilities (14). Pehlivan Sarıbudak et al. (15) reported that sustained organizational stress was associated with lower psychological resilience, even among individuals who initially exhibited strong adaptation mechanisms. Furthermore, a study by Zhai et al. (16) reported associations between stress factors and functional decline. Taken together, the above evidence suggests close interconnections among fatigue, stress, and psychological resource capacity in nurses.

Nurses working in critical care units who experience sleep concerns report higher levels of fatigue, cognitive decline, and burnout (17). This pattern is particularly pronounced among individuals working long night shifts, where disruption of normal circadian rhythms has been associated with metabolic disorders and depressive symptoms (18). During the COVID-19 pandemic, frontline nurses also reported severe sleep fragmentation and heightened anxiety alongside poorer physical and mental health (19). While these studies are intriguing, evidence concerning the associations of worries about sleep outcomes and difficulty falling asleep with fatigue and stress among nurses in general care units remains limited.

Existing research exploring these associations struggles to capture the complex and dynamic interrelationships among these factors (20). The current study integrated network analysis and latent profile analysis (LPA). The network analysis approach facilitated development of a model illustrating the interconnections among mental health promotion, sleep concerns, fatigue, and stress. Based on the JD-R-informed framework, we hypothesized that (H1) mental health promotion would be negatively associated with fatigue and perceived stress, and (H2) sleep concerns would be positively associated with fatigue and perceived stress. We further hypothesized that (H3) higher mental health promotion and lower sleep concerns would be associated with a greater likelihood of membership in latent profiles characterized by relatively lower levels of fatigue and perceived stress. Furthermore, LPA was used to identify subgroups with different combinations of fatigue and stress dimensions. This approach may inform the identification of relevant symptom patterns and subgroups for future occupational-health research and planning. Network analysis can be used to explore the interactions between psychological and behavioral variables at the subdimension level, while LPA can identify population heterogeneity based on patterns of fatigue and stress. This study integrates these two methods, providing both a micro-level understanding of the relationships between variables and a macro-level classification of subgroups, thus offering a more comprehensive research perspective than traditional association analysis.

## 2 Methods

### 2.1 Study design

This multicenter cross-sectional survey was conducted among clinical nurses from three tertiary hospitals in Anhui Province, China. The study is reported in accordance with the Strengthening the Reporting of Observational Studies in Epidemiology (STROBE) statement for cross-sectional studies (Supplementary File S1).

### 2.2 Participants

Because the sample-size requirements for network analysis and latent profile analysis depend on different model-specific characteristics, no single established *a priori* formula was available to determine the required sample size for both analyses simultaneously. During the prespecified data-collection period, questionnaires were distributed through the nursing administration departments of the three participating hospitals to 352 eligible and accessible nurses. This number reflected the nurses who met the eligibility criteria and could be recruited during the study period, rather than a target sample size generated directly from a single statistical formula. Eligible participants were clinical nurses employed at one of the three participating tertiary hospitals, had at least 1 year of clinical experience, and provided written informed consent. Nurses with less than 1 year of clinical experience or who did not provide consent were not eligible. Returned questionnaires were excluded from analysis if one or more required items were incomplete or if the attention-check item was answered incorrectly. After 14 questionnaires that did not meet the prespecified validity criteria were excluded, 338 participants were included in the final analyses. *Post hoc* sensitivity and stability assessments based on the final analytic sample are described in the Statistical analyses section. The present report focuses on questionnaire data; concurrently collected heart rate variability data were not included in the current analyses.

### 2.3 Data collection procedure

Data were collected between January and June 2025 using paper questionnaires distributed through the nursing administration departments of the participating hospitals. After receiving information about the study and providing written informed consent, participants completed the questionnaires independently. Returned questionnaires were screened according to the prespecified completeness and attention-check criteria, and valid responses were entered into an electronic database for analysis.

### 2.4 Measures

#### 2.4.1 Mental health promotion scale (MHPS)

This study used the Chinese version of the Mental Health Promotion Scale (MHPS) to assess mental health promotion ability. The scale was originally designed and proposed by Kadioglu et al. (21) and was culturally adapted and validated by Hou et al. (22) among clinical nurses in China. The MHPS is composed of 12 dimensions: friendship, values, personal development, sexual behavior, self-esteem, stress coping, physical health, family and intimate relationships, communication, ability to refuse, emotional control, and self-awareness, with a total of 39 items. All items were scored using a 5-point Likert scale, with higher scores indicating stronger mental health promotion ability. The Chinese version of the MHPS showed good internal consistency, with a Cronbach’s *α* coefficient of 0.961 for the total scale; in this study, the Cronbach’s α coefficient was 0.911.

#### 2.4.2 Anxiety and preoccupation about sleep questionnaire (APSQ)

The APSQ was originally developed by Tang and Harvey (23) for patients with self-reported sleep-related worries about insomnia or related sleep disorders. Jansson-Fröjmark et al. (24) revised the scale to include two dimensions: sleep-related anxiety (6 items) and sleep-related preoccupation (4 items). Shi et al. (25) translated and validated a Chinese version of the APSQ suitable for shift nurses. The items were scored using a 5-point Likert scale, ranging from 1 (“strongly disagree”) to 5 (“strongly agree”), with a total score ranging from 10 to 50; higher scores indicated greater sleep concerns. Throughout this manuscript, “sleep concerns” refers to the overall APSQ construct, whereas “sleep-related anxiety” (APSQ1) and “sleep-related preoccupation” (APSQ2) refer to its two subdimensions. The scale showed good internal consistency, with the Cronbach’s *α* value of the Chinese version of the APSQ reported as 0.88. In the present sample, Cronbach’s α was 0.919 for the total APSQ, 0.861 for the sleep-related anxiety subscale, and 0.742 for the sleep-related preoccupation subscale.

#### 2.4.3 Multidimensional fatigue inventory (MFI)

The Chinese version of MFI was used to assess fatigue. This scale was originally developed by Smets et al. (26) and was subsequently validated in the Chinese version by Miao et al. (27). The MFI comprises five subscales: general fatigue, physical fatigue, mental fatigue, decreased motivation, and reduced activity, with four items in each subscale, resulting in a total of 20 items. Participants used a 5-point Likert scale to assess their fatigue experience over the past week, ranging from 1 (“No, this is not true”) to 5 (“Yes, this is true”), with higher scores indicating more severe fatigue symptoms. In this study, the Cronbach’s *α* coefficient was 0.926.

#### 2.4.4 Chinese version of the perceived stress scale (CPSS)

The CPSS was used to measure perceived stress. This scale was originally developed by Cohen et al. (28) and then subsequently translated and validated for the Chinese population by Yang (29). All items were scored using a 5-point Likert scale, ranging from 0 (“never”) to 4 (“very frequently”), with higher scores indicating higher levels of perceived stress. In this study, the Cronbach’s alpha coefficient of the CPSS was 0.934.

### 2.5 Statistical analyses

Statistical analyses were performed using SPSS version 26.0. Continuous variables were summarized as mean ± standard deviation (SD) or median and interquartile range, as appropriate to their distributions, and categorical variables were summarized as counts and percentages. Before analysis, the final analytic dataset was examined for missing values; no imputation or other missing-data procedure was required. Age and years of work experience were categorized for inferential analyses to facilitate interpretation across career stages and avoid assuming linear associations with profile membership. All tests were two-sided, with *p* < 0.05 considered statistically significant.

Network analysis was conducted in R version 4.5.1 (30). The bootnet package (version 1.5.6) was used for network estimation, bootstrap confidence intervals for edge weights, and case-dropping centrality-stability analysis; qgraph (version 1.9.8) was used for network visualization and centrality indices (31); and mgm (version 1.2.12) was used to calculate node predictability (32). Centrality indices included strength, closeness, and betweenness (33), and bridge centrality was calculated using functions from networktools. Bridge strength was defined as the sum of the absolute edge weights connecting a node to nodes in other construct communities (34). Scale subdimensions rather than individual items were used as nodes, yielding a 21-node network comprising 12 MHPS, 2 APSQ, 5 MFI, and 2 CPSS subdimensions. An item-level model would have included 83 nodes and up to 3,403 possible pairwise edges, substantially increasing model complexity and potential estimation instability. After data collection, a *post hoc* correlation-based sensitivity analysis was conducted for the 21-node network to estimate the detectable zero-order correlation threshold in the achieved sample; the result is reported in the Results. This analysis was not treated as a complete power analysis for conditional network-edge or latent-profile recovery.

LPA was performed in Mplus 8.0 using the five MFI and two CPSS subdimensions as profile indicators. The optimal number of profiles was evaluated using the Akaike Information Criterion (AIC), Bayesian Information Criterion (BIC), adjusted Bayesian Information Criterion (aBIC), entropy, class proportions, average posterior classification probabilities, the bootstrap likelihood ratio test (BLRT), and the Lo–Mendell–Rubin adjusted likelihood ratio test (LMR) (35). Lower AIC, BIC, and aBIC values indicated better relative fit, whereas higher entropy and average posterior probabilities indicated better within-sample classification quality; these measures were not interpreted as evidence of profile stability in an independent sample. AIC, BIC, aBIC, class proportions, entropy, and average posterior classification probabilities were independently verified in R 4.5.1, whereas BLRT and LMR *p* values were obtained from Mplus 8.0.

After the optimal profile solution was selected, categorical variables were compared across profiles using Pearson’s chi-squared test or Fisher’s exact test, as appropriate, and continuous scale scores were compared using one-way analysis of variance. Variables associated with profile membership were subsequently examined using ridge-penalized multinomial logistic regression. The penalty parameter was selected by stratified five-fold cross-validation, and uncertainty was quantified using 500 stratified bootstrap samples. Before multivariable modelling, candidate demographic predictors were assessed for collinearity and redundancy; when two predictors showed substantial overlap, only one was retained based on interpretability and model stability.

## 3 Results

### 3.1 Participant flow and characteristics

All 352 questionnaires distributed to eligible nurses were returned. Fourteen questionnaires were excluded because one or more required items were incomplete or the attention-check item was answered incorrectly, leaving 338 valid questionnaires for analysis. The questionnaire return rate was 100%, and the valid-response rate was 96.0%. No missing values were present in the final analytic dataset. In the *post hoc* sensitivity analysis, with n = 338, two-sided *α* = 0.05, 80% power, and Bonferroni correction across 210 possible pairwise associations, the 21-node network could detect correlations of approximately |r| ≥ 0.242. Of these participants, 311 (92.0%) were female and 27 (8.0%) were male. The median age was 34 years (IQR, 29–38; observed range, 20–58 years), and the median years of work experience was 11 years (IQR, 6–15; observed range, 1–37 years; valid n = 338).

### 3.2 Associations among mental health promotion, sleep concerns, fatigue, and stress networks

**Figure 1:**
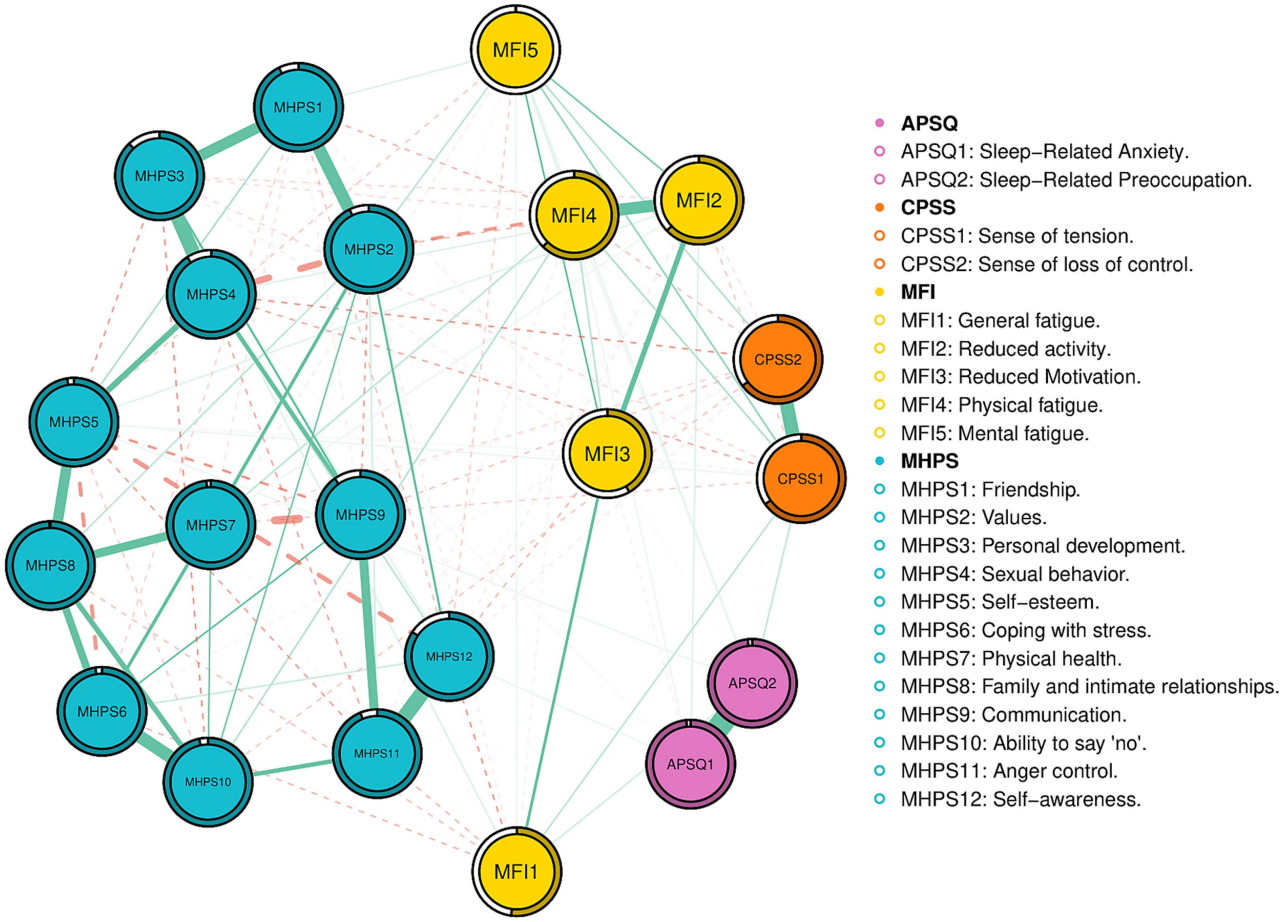
Network diagram of nurses’ mental health promotion, sleep concerns, fatigue, and stress; edge colors indicate the sign of the association, with positive associations represented by solid green lines; edge thickness represents association strength, with thicker lines representing stronger associations; concentric rings surrounding each node represent the proportion of variance predicted by neighboring nodes.

In the current study, the extended BIC was used for model selection, and the hyperparameter was adjusted to 0.5 for network analysis (see Figure 1). No redundant variables were removed from the model, and all nodes exhibited significantly different correlation patterns with other nodes (36). For the fatigue dimension, the predictability values for general fatigue (MFI1), reduced activity (MFI2), decreased motivation (MFI3), and physical fatigue (MFI4) were 0.521, 0.629, 0.418, and 0.624, respectively, indicating that adjacent nodes explained 52, 63, 42, and 62% of variance, respectively. For perceived stress, the predictability values for sense of tension (CPSS1) and sense of loss of control (CPSS2) were 0.652 and 0.648, respectively, indicating that approximately 65% of its variance can be explained by neighboring nodes. The average predictability of all nodes was 0.79, indicating that 79% of the variance among nodes could be explained by network connectivity. The final network contained 21 nodes and 53 non-zero edges, with an edge density of 0.252. Among these edges, some of the strongest positive and negative connections involved MHPS2 (values). Specifically, MHPS2 (values) was positively correlated with MHPS1 (friendship; edge weight = 0.60) and negatively correlated with MFI4 (physical fatigue; edge weight = −0.17). Most negative edges involve decreased motivation (MFI3) and a sense of loss of control (CPSS2). Decreased motivation (MFI3) was significantly negatively correlated with APSQ1 (sleep-related anxiety; edge weight = −0.01), MHPS1 (friendship; edge weight = −0.01), MHPS3 (personal development; edge weight = −0.01), and MHPS12 (self-awareness; edge weight = −0.06). Furthermore, CPSS2 (sense of loss of control) was also negatively correlated with MHPS4 (sexual behavior; edge weight = −0.01), MHPS6 (coping with stress; edge weight = −0.01), and MHPS7 (physical health; edge weight = −0.01). APSQ1 (sleep-related anxiety) was positively correlated with MFI1 (general fatigue; edge weight = 0.02), and APSQ2 (sleep-related preoccupation) was positively correlated with CPSS1 (sense of tension; edge weight = 0.03). MFI5 (mental fatigue) was positively correlated with CPSS1 (sense of tension; edge weight = 0.06). See Figure 1 for more details.

**Figure 2:**
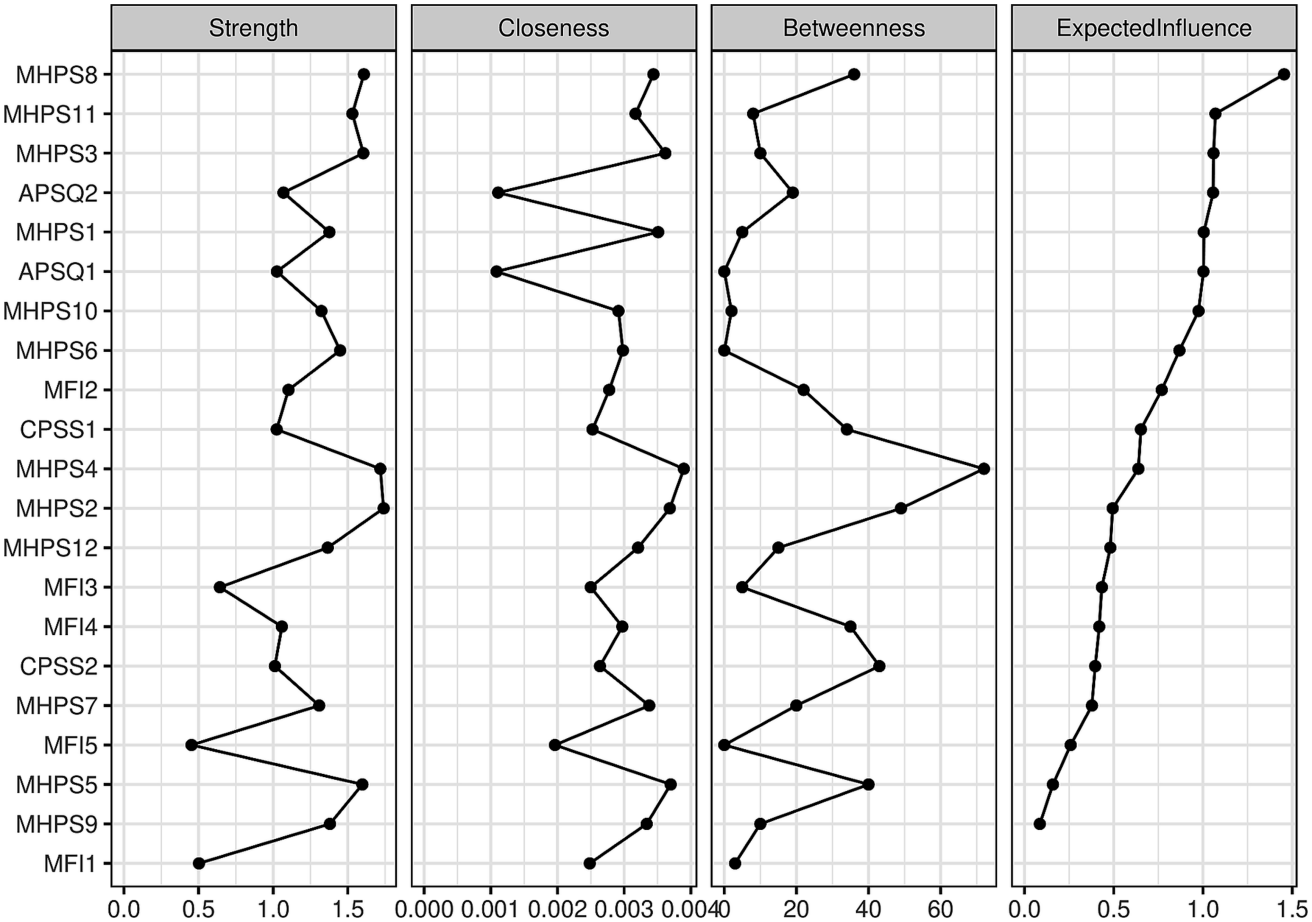
Centrality indicators of all nodes within the network.

Figure 2 illustrates the normalized centrality indices of all nodes. Within the network structure, MHPS2 (values) exhibited the highest strength, whereas MHPS4 (sexual behavior) had the highest closeness and betweenness values. These indices describe the node’s topological position in the estimated network and should not be interpreted as evidence of a mechanism, causal influence, or clinical importance. Furthermore, other mental health promotion domains—including MHPS3 (personal development), MHPS5 (self-esteem), and MHPS8 (family and intimate relationships)—also demonstrated relatively high centrality values, indicating relatively central topological positions within the estimated network. Regarding fatigue and perceived stress, the centrality indices of MFI2 (reduced activity) and MFI4 (physical fatigue) were higher than those of the perceived stress dimension, reflecting greater topological connectedness rather than causal influence.

**Figure 3:**
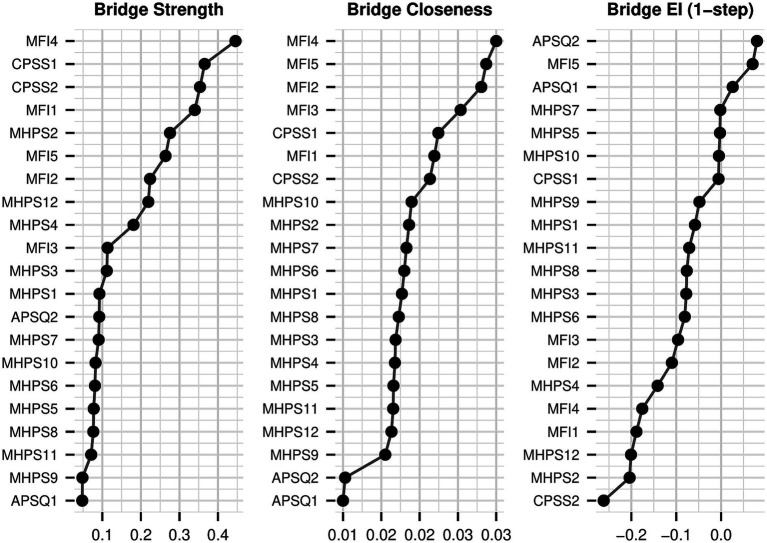
Bridging centrality index of all nodes within the network.

**Figure 4:**
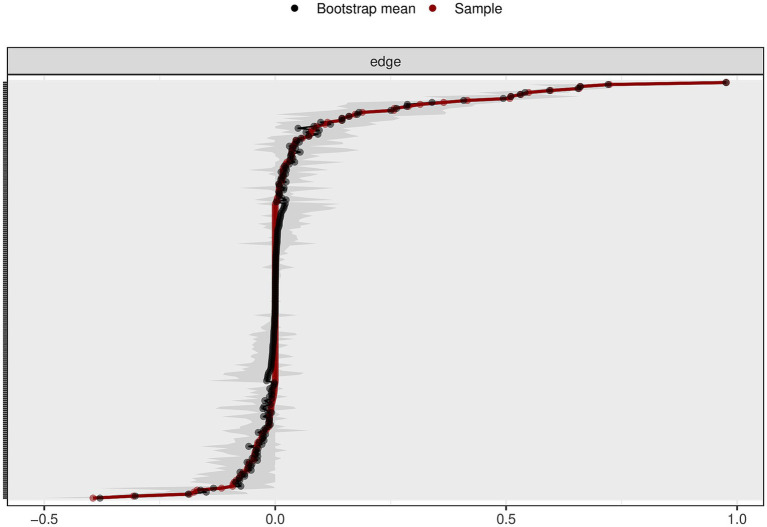
Accuracy analysis of edge weights within the network of mental health promotion, sleep concerns, fatigue, and stress; average edge weights across bootstrap samples are represented by black lines, and edge weights in the study sample are represented by red lines.

**Figure 5:**
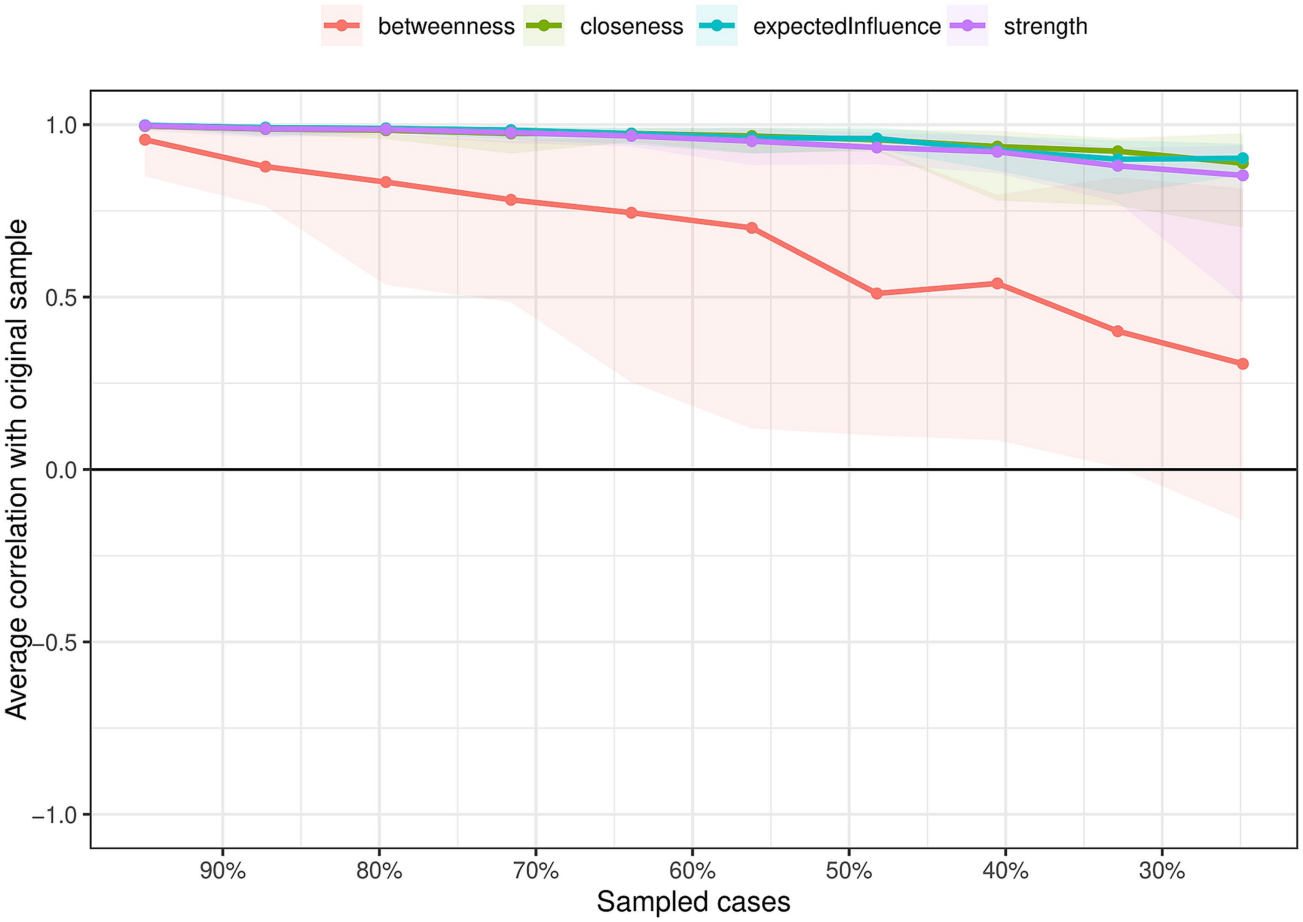
Estimation of the stability of the network structure.

Bridging centrality indicators showed that MFI4 (physical fatigue), CPSS1 (sense of tension), and APSQ2 (sleep-related preoccupation) were key bridge nodes connecting different construct communities (Figure 3). MFI4 (physical fatigue) had the highest bridge strength value (0.445), while CPSS1 (sense of tension) and CPSS2 (sense of loss of control) had values of 0.365 and 0.353, respectively. MFI4 (physical fatigue) and the stress-related dimensions occupied bridge positions connecting different construct communities. MFI4 (physical fatigue) also had the highest bridge closeness value (0.030), followed by MFI5 (mental fatigue; 0.029) and MFI2 (reduced activity; 0.028), indicating that these nodes were central in terms of proximity to other nodes. APSQ2 (sleep-related preoccupation) had the highest bridge expected influence (EI) value (0.080), while MFI5 (mental fatigue; 0.070) and APSQ1 (sleep-related anxiety; 0.026) also showed comparatively high bridge expected influence values. These indices describe network topology and do not imply effects on other constructs. The case-dropping bootstrap analysis showed correlation-stability coefficients of 0.75 for strength centrality and 0.75 for closeness centrality, indicating good stability for these two indices. In contrast, the correlation-stability coefficient for betweenness centrality was 0.13, indicating limited stability; therefore, betweenness rankings were interpreted cautiously. Figures 4, 5 show the bootstrap confidence intervals of the edge weights and the stability plots of the centrality index, respectively.

### 3.3 Potential characteristics of fatigue/stress

**Figure 6:**
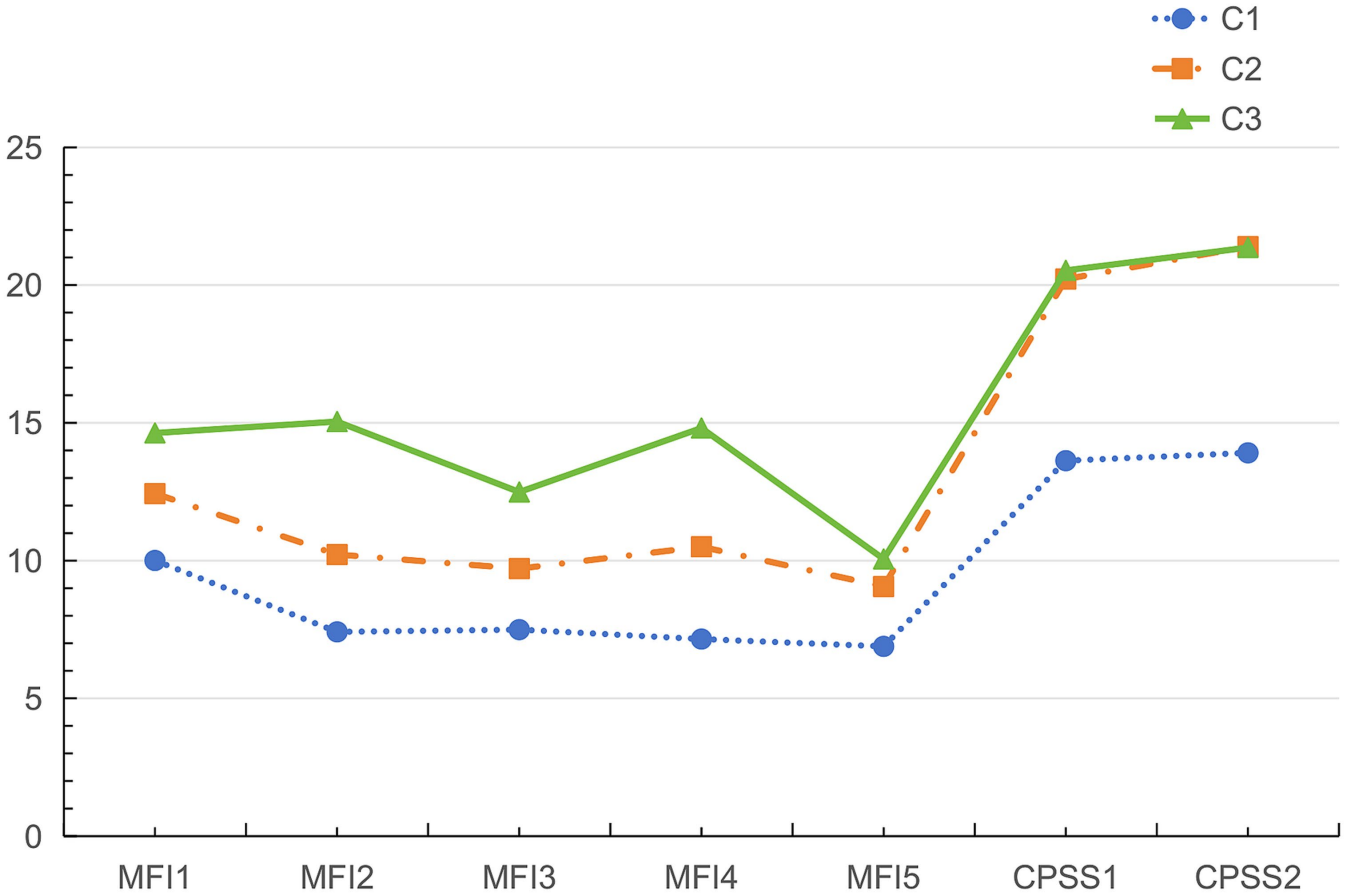
Three latent profiles of nurse fatigue and stress; C1, low fatigue/low stress profile; C2, moderate fatigue/high stress profile; C3, high fatigue/high stress profile.

The current study used five subdimension scores from the MFI and two subdimension scores from the CPSS as variables to construct latent profile models. By gradually increasing the number of profiles, one to four-profile models were compared. Fit indices of the different models are detailed in Table 1. As the number of latent profiles increased, the AIC, BIC, and aBIC indices gradually decreased, indicating a corresponding improvement in model fit. When the number of profiles was three, entropy was 0.884, BLRT was significant (*p* < 0.001), and the LMR test was significant (*p* = 0.0452). Although the four-profile model had lower information criteria, its LMR test was not significant (*p* = 0.2111), entropy decreased to 0.853, and its two smallest profiles contained only 6.2 and 5.9% of participants. Therefore, this study selected the three-profile model (C1, C2, and C3) of fatigue and stress among clinical nurses for in-depth analysis. Figure 6 shows the score distribution of each subdimension of fatigue and stress in the three profiles. Profile 1 included 33 participants (9.8%), whose scores in all subdimensions of fatigue and stress were low. Therefore, this profile was named the “low fatigue/low stress group.” Although this subgroup contained a relatively small number of participants, it represented a distinct low-risk pattern and had an average posterior classification probability of 0.898. Profile 2, comprising 241 participants (71.3%), showed moderate scores on the fatigue subdimensions and high scores on the stress subdimensions; this group was designated as the “moderate fatigue/high stress group,” with an average posterior probability of 0.958. Profile 3, consisting of 64 participants (18.9%), scored higher on most dimensions than the other two groups; this profile was identified as the “high fatigue/high stress group,” with an average posterior probability of 0.950. Overall, the distinct characteristics and high average posterior probabilities of the three profiles support the within-sample interpretability and classification quality of the model and indicate model-based heterogeneity in fatigue and stress response patterns among clinical nurses. Because no external or split-sample replication was conducted, profile stability beyond this sample cannot be claimed. These profiles should be interpreted as statistically derived, model-based classifications of response patterns rather than as naturally occurring or externally validated psychological subpopulations.

**Table 1:** Fit indices and classification quality of candidate latent profile models of nurse fatigue and stress (*n* = 338).

Model	AIC	BIC	aBIC	Entropy	BLRT P	LMR P	Class proportions	Smallest class, *n* (%)	Average posterior probabilities
1	12091.982	12145.504	12101.094	—	—	—	1.000	338 (100.0%)	1.000
2	11754.604	11838.711	11768.923	0.821	<0.001	0.0051	0.749/0.251	85 (25.1%)	0.957/0.922
3	11560.567	11675.259	11580.094	0.884	<0.001	0.0452	0.098/0.713/0.189	33 (9.8%)	0.898/0.958/0.950
4	11438.187	11583.462	11462.920	0.853	<0.001	0.2111	0.062/0.589/0.290/0.059	20 (5.9%)	0.924/0.925/0.870/0.981

AIC, Akaike information criterion; BIC, Bayesian information criterion; aBIC, sample-size-adjusted Bayesian information criterion; BLRT, bootstrap likelihood ratio test; LMR, Lo–Mendell–Rubin adjusted likelihood ratio test. AIC, BIC, aBIC, class proportions, smallest-class sizes, entropy, and average posterior probabilities were verified in the independent R 4.5.1 re-estimation; BLRT and LMR *p* values were obtained from Mplus 8.0.

### 3.4 Sleep concerns and mental health promotion across the three profiles

Univariate analysis showed statistically significant differences among the three groups in terms of gender, age, years of work experience, employment type, mental health promotion score, and APSQ total score (*p* < 0.05). Detailed results are shown in Tables 2, 3.

**Table 2:** General demographic information of participants and results of univariate analysis across three latent fatigue/stress profiles.

Demographic variables		Total number (*n* = 338)	Low fatigue/low stress profile (*n* = 33)	Moderate fatigue/high stress profile (*n* = 241)	High fatigue/high stress profile (*n* = 64)	Statistical value	*p-*value
Sex	Male	27	6 (18.2)	12 (5.0)	9 (14.1)	10.847 ^a)^	0.004
	Female	311	27 (81.8)	229 (95.0)	55 (85.9)		
Age	< 25	18	3 (9.1)	11 (4.6)	4 (6.2)	16.746 ^b)^	0.002
	25–45	301	24 (72.7)	223 (92.5)	54 (84.4)		
	> 45	19	6 (18.2)	7 (2.9)	6 (9.4)		
Years of work experience	< 5	71	6 (18.2)	47 (19.5)	18 (28.1)	15.667 ^b)^	0.004
	5–25	248	21 (63.6)	186 (77.2)	41 (64.1)		
	> 25	19	6 (18.2)	8 (3.3)	5 (7.8)		
Education level	Junior College	32	6 (18.2)	21 (8.7)	5 (7.8)	8.964 ^b)^	0.068
	Bachelor’s Degree	292	27 (81.8)	206 (85.5)	59 (92.2)		
	Postgraduate	14	0 (0.0)	14 (5.8)	0 (0.0)		
Professional title	Senior nurse	143	12 (36.4)	100 (41.5)	31 (48.4)	3.055 ^b)^	0.655
	Head nurse	176	20 (60.6)	125 (51.9)	31 (48.4)		
	Deputy chief nurse	19	1 (3.0)	16 (6.6)	2 (3.1)		
Self-reported physical health status	Poor	41	1 (3.0)	26 (10.8)	14 (21.9)	9.410 ^a)^	0.052
	Fair	205	24 (72.7)	146 (60.6)	35 (54.7)		
	Good	92	8 (24.2)	69 (28.6)	15 (23.4)		
Exercise habits	No	125	7 (21.2)	96 (39.8)	22 (34.4)	4.549 ^a)^	0.103
	Yes	213	26 (78.8)	145 (60.2)	42 (65.6)		
Hobby	No	96	6 (18.2)	74 (30.7)	16 (25.0)	2.688 ^a)^	0.261
	Yes	242	27 (81.8)	167 (69.3)	48 (75.0)		
Marital status	Unmarried	76	9 (27.3)	51 (21.2)	16 (25.0)	0.908 a)	0.635
	Married	262	24 (72.7)	190 (78.8)	48 (75.0)		
Parity	No Children	98	9 (27.3)	68 (28.2)	21 (32.8)	0.572 ^a)^	0.751
	Children	240	24 (72.7)	173 (71.8)	43 (67.2)		
Alcohol/coffee consumption	No	222	22 (66.7)	160 (66.4)	40 (62.5)	0.355 ^a)^	0.837
	Yes	116	11 (33.3)	81 (33.6)	24 (37.5)		
Night shifts in the past month	No	146	19 (57.6)	106 (44.0)	21 (32.8)	5.654 ^a)^	0.059
	Yes	192	14 (42.4)	135 (56.0)	43 (67.2)		
Self-reported diseases	No	226	22 (66.7)	162 (67.2)	42 (65.6)	0.059 ^a)^	0.971
	Yes	112	11 (33.3)	79 (32.8)	22 (34.4)		
Employment type	Contract	275	24 (72.7)	200 (83.0)	51 (79.7)	19.938 ^b)^	0.002
	Personnel agency	39	1 (3.0)	31 (12.9)	7 (10.9)		
	Permanent staff	24	8 (24.2)	10 (4.1)	6 (9.4)		

a) Pearson chi-squared test; b) Fisher’s exact test.

**Table 3:** Results of univariate analysis of sleep concerns and mental health promotion scales across the three latent fatigue/stress profiles.

Scale	Low fatigue/low stress profile (*n* = 33)	Moderate fatigue/high stress profile (*n* = 241)	High fatigue/high stress profile (*n* = 64)	*F* value	*p-*value
Sleep concerns	18.12 ± 8.19	28.15 ± 11.96	30.97 ± 12.22	13.725	<0.001
Mental health promotion	147.42 ± 15.26	128.23 ± 7.16	113.22 ± 10.28	166.534	<0.001

Values are presented as mean ± SD. Between-profile differences were tested using one-way analysis of variance.

### 3.5 Factors associated with fatigue/stress profiles

Age and years of work experience showed substantial overlap (r = 0.94); therefore, age was retained for interpretability and years of work experience was excluded from the final multivariable model. Cross-validated ridge multinomial logistic regression was then used with the “low fatigue/low stress” profile (C1) as the reference category (Tables 4, 5). For C2 versus C1, higher sleep concerns were associated with higher odds of C2 membership [OR = 1.060, bootstrap 95% CI (1.042, 1.078), *p* < 0.001], whereas higher mental health promotion [OR = 0.921, bootstrap 95% CI (0.905, 0.944), *p* < 0.001] and male sex (OR = 0.323, bootstrap 95% CI [0.131, 0.840], *p* = 0.014) were associated with lower odds. Personnel agency employment was associated with higher odds of C2 membership relative to contract employment [OR = 2.754, bootstrap 95% CI (1.480, 5.160), *p* = 0.003]. For C3 versus C1, higher sleep concerns were associated with higher odds of C3 membership [OR = 1.062, bootstrap 95% CI (1.038, 1.085), *p* < 0.001], whereas higher mental health promotion was associated with lower odds [OR = 0.803, bootstrap 95% CI (0.772, 0.831), *p* < 0.001]. Other demographic variables were not statistically significant in the C3-versus-C1 comparison.

**Table 4:** Cross-validated ridge multinomial logistic regression estimates for membership in the moderate fatigue/high stress profile (C2) versus the low fatigue/low stress profile (C1).

Predictor	*B*	Bootstrap *SE*	*Z*	*p*-value	OR	Bootstrap 95% CI
Sleep concerns	0.058	0.009	6.493	<0.001	1.060	[1.042, 1.078]
Mental health promotion	−0.082	0.011	−7.622	<0.001	0.921	[0.905, 0.944]
Male	−1.130	0.460	−2.457	0.014	0.323	[0.131, 0.840]
Age 25–45 years	−0.039	0.378	−0.102	0.919	0.962	[0.457, 1.988]
Age >45 years	−0.699	0.738	−0.948	0.343	0.497	[0.115, 2.025]
Personnel agency	1.013	0.339	2.989	0.003	2.754	[1.480, 5.160]
Permanent staff	−0.768	0.522	−1.470	0.142	0.464	[0.173, 1.317]

Estimates represent the odds of membership in C2 (moderate fatigue/high stress) relative to C1 (low fatigue/low stress). Female sex, age <25 years, and contract employment were the reference categories. Estimates were obtained using cross-validated ridge multinomial logistic regression (*n* = 338; lambda = 0.02707). SE, standard error; OR, odds ratio; CI, confidence interval. Bootstrap SEs, Z statistics, *p*-values, and 95% CIs were estimated using 500 stratified bootstrap samples.

**Table 5:** Cross-validated ridge multinomial logistic regression estimates for membership in the high fatigue/high stress profile (C3) versus the low fatigue/low stress profile (C1).

Predictor	*B*	Bootstrap *SE*	*Z*	*p*-value	OR	Bootstrap 95% CI
Sleep concerns	0.061	0.012	5.161	<0.001	1.062	[1.038, 1.085]
Mental health promotion	−0.219	0.019	−11.524	<0.001	0.803	[0.772, 0.831]
Male	0.169	0.529	0.320	0.749	1.184	[0.476, 3.642]
Age 25–45 years	−0.938	0.652	−1.439	0.150	0.392	[0.114, 1.271]
Age >45 years	−0.126	0.814	−0.155	0.877	0.882	[0.183, 4.405]
Personnel agency	0.802	0.437	1.833	0.067	2.230	[0.928, 5.105]
Permanent staff	−0.234	0.610	−0.383	0.702	0.792	[0.238, 2.862]

Estimates represent the odds of membership in C3 (high fatigue/high stress) relative to C1 (low fatigue/low stress). Female sex, age <25 years, and contract employment were the reference categories. Estimates were obtained using cross-validated ridge multinomial logistic regression (*n* = 338; lambda = 0.02707). SE, standard error; OR, odds ratio; CI, confidence interval. Bootstrap SEs, Z statistics, *p*-values, and 95% CIs were estimated using 500 stratified bootstrap samples.

## 4 Discussion

### 4.1 Association among mental health promotion, sleep concerns, fatigue, and stress

From a JD-R-informed perspective, the negative associations of mental health promotion with fatigue and stress were consistent with a resource-strain pattern, whereas the positive associations of sleep concerns with fatigue and stress were consistent with a burden-strain pattern. Given the cross-sectional design, these associations should not be interpreted as evidence of causal pathways. This interpretation was intended to contextualize the observed resource-strain and burden-strain associations, rather than to claim that the present cross-sectional data provide a comprehensive test of JD-R processes. The current results indicate that occupational values (MHPS2) are a core psychological resource and that sleep-related preoccupation (APSQ2) has strong connections with other nodes in the fatigue/stress network of clinical nurses. Network analysis showed that several dimensions of mental health promotion—values (MHPS2), personal development (MHPS3), and friendship (MHPS1)—were negatively correlated with fatigue and stress, while the two dimensions of sleep concerns—sleep-related anxiety (APSQ1) and sleep-related preoccupation (APSQ2)—were positively correlated with fatigue and stress. The average node predictability was 0.79, indicating that a substantial proportion of node variance was accounted for by neighboring nodes. The case-dropping bootstrap supported the stability of strength and closeness centrality (CS coefficients = 0.75 for both), whereas betweenness centrality was less stable (CS coefficient = 0.13) and was interpreted cautiously. Values (MHPS2) showed the highest strength centrality. Bridge indices further indicated that physical fatigue (MFI4), sense of tension (CPSS1), and sleep-related preoccupation (APSQ2) occupied relatively central bridge positions connecting the mental health promotion, sleep-concern, and fatigue/stress communities. To avoid overinterpretation of the psychological network findings, edges, centrality indices, and bridge indices should be interpreted as regularized conditional associations and relative topological positions within this sample-specific network, rather than as causal pathways, psychological mechanisms, clinical importance, or confirmed intervention targets. This is consistent with studies showing that mental health promotion is associated with lower occupational stress and better sleep quality (37, 38), possibly because sleep concerns are closely associated with higher fatigue and psychological distress (39, 40), and mental health promotion is associated with lower fatigue and related symptoms caused by shift work and high-intensity work demands (41). Through LPA, three statistically derived fatigue/stress profiles—low fatigue/low stress, moderate fatigue/high stress, and high fatigue/high stress—were identified, indicating provisional, model-based fatigue/stress response patterns in this sample rather than evidence of true latent subpopulations. This echoes previous research on the heterogeneity of perceived stress and work engagement among clinical nurses (42). The ridge multinomial results further showed that mental health promotion and sleep concerns consistently distinguished both the moderate fatigue/high stress and high fatigue/high stress profiles from the low fatigue/low stress profile. Higher mental health promotion was associated with lower odds of membership in C2 and C3, whereas higher sleep concerns were associated with higher odds of membership in both profiles. These results highlight the interrelationships among psychological resources, sleep concerns, and fatigue/stress patterns (43, 44). Mental health promotion is an important psychological resource that may be associated with better coping with work demands, lower sleep-related anxiety, and lower levels of fatigue and stress (45). These findings provide hypotheses for future longitudinal and intervention research examining psychological resources, sleep concerns, and fatigue/stress patterns.

### 4.2 Associations of mental health promotion dimensions within the fatigue/stress network

Occupational values are associated with lower levels of fatigue and stress under high-intensity work demands and may represent a core psychological resource in clinical practice (46). These values differ from stable personality traits, in that they are the result of social construction and professional training, rather than innate individual characteristics (47). Network analysis showed that multiple mental health promotion dimensions were negatively associated with nodes in the fatigue/stress network. Among them, values (MHPS2) was found to be the core dimension, reflecting the unique value-driven characteristics of the nursing profession. Nursing is not just a series of technical tasks, but a valuable practice that is deeply rooted in care for human life, promotion of health, and alleviation of suffering. Occupational values, such as dedication, compassion, responsibility, and the sanctity of life, are core motivations for nurses to choose nursing as a profession (48). This study found that values (MHPS2) were positively correlated with friendship (MHPS1) and negatively correlated with physical fatigue (MFI4). These results indicate that higher values scores were associated with stronger friendship scores and lower physical-fatigue scores. This pattern is consistent with the qualitative research reported by Wang et al. (49), which linked organizational atmosphere and meaning in work with values and nurses’ mental health; however, professional meaning was not directly measured in the present study.

Sexual behavior (MHPS4) showed the highest closeness and betweenness centralities; however, the betweenness estimate had limited stability, and centrality alone does not demonstrate a clinical mechanism. Recovery experience theory has discussed psychological detachment, relaxation, and positive emotions outside work (50), but this literature provides only broader context. Sexual behavior (MHPS4) was negatively correlated with sense of loss of control (CPSS2), but the direct edge was very small (edge weight = −0.01). Broader quality-of-life research has considered the private sphere in relation to nurses’ mental health (51), but the present study did not assess recovery, intimacy, perceived control, or related mechanisms; therefore, none can be inferred from this edge. Thus, the MHPS4 centrality finding should be regarded as exploratory and requires independent replication. Multiple dimensions of mental health promotion, including personal development (MHPS3), self-esteem (MHPS5), friendship (MHPS1), coping with stress (MHPS6), and physical health (MHPS7), showed negative correlations with fatigue and stress-related symptoms, such as decreased motivation (MFI3) and sense of loss of control (CPSS2). These correlations indicate a broad pattern of associations rather than evidence that the resources operate synergistically. The negative correlation between friendship and decreased motivation echoes findings from studies on engagement in social connection promotion (52), while the negative correlation between coping with stress (MHPS6) and sense of loss of control (CPSS2) is consistent with studies on the moderating role of coping styles between psychological capital and psychological stress among nurses (53).

LPA and ridge multinomial regression results further supported the pattern observed in the network analysis. The total score for mental health promotion differentiated fatigue/stress heterogeneity: higher mental health promotion was associated with lower odds of membership in both the moderate fatigue/high stress and high fatigue/high stress profiles relative to the low fatigue/low stress profile. This finding is consistent with the findings of Mao et al. (54), who reported that interventions enhancing psychological resilience reduced stress among healthcare workers. The present results suggest that mental health promotion, particularly factors related to intrinsic value systems and quality of life, is inversely associated with fatigue/stress profiles. Future longitudinal or intervention studies could evaluate whether health-promotion programs that address professional values, interpersonal relationships, coping skills, and work-life balance are followed by changes in resilience, fatigue, and stress.

### 4.3 Sleep concerns as bridge nodes in the fatigue/stress network

The current study examined the bridging positions of sleep concerns, comprising sleep-related anxiety (APSQ1) and sleep-related preoccupation (APSQ2), in the fatigue/stress network of clinical nurses. Sleep concerns are prevalent among nurses and constitute a core health challenge (55). Network analysis revealed that sleep-related preoccupation (APSQ2) was a prominent bridge node, with the highest bridge expected influence. Sleep-related preoccupation was associated with both stress and fatigue-related dimensions. These sleep-related cognitions may be evaluated in future studies of nurse-centered sleep education or cognitive behavioral therapy for insomnia (CBT-I) (56), which can be brief and flexible to accommodate shift work and irregular schedules. Sleep-related preoccupation (APSQ2) was also positively correlated with sense of tension (CPSS1). Interpreting this association in terms of perceived sleep control is theoretically plausible, but perceived control and the proposed cognitive process were not independently measured. These results are consistent with the study by Lai et al. (57), which found links among stress, sleep quality, anxiety, and depressive symptoms. The network pattern further suggests that sleep-related preoccupation was highly connected with anxiety and stress-related dimensions; however, temporal direction was not assessed. Sleep-related anxiety (APSQ1) was positively correlated with overall fatigue (MFI1), indicating that nurses’ anticipation and fear of negative consequences of sleep deprivation, such as insufficient energy, work errors, or deteriorating health the following day, was positively associated with overall fatigue. This result echoes the findings of Qin et al. (58), who found that sleep quality plays a mediating role between work stress and depression levels. Sleep-related anxiety (APSQ1) was also negatively correlated with decreased motivation (MFI3), suggesting that persistent sleep-related anxiety may be theoretically related to lower motivation. This echoes the qualitative findings of Azizoddin et al. (59), who found that “psychological stress” and “lack of motivation” were the main causes of fatigue. LPA results showed significant differences in APSQ scores among the three fatigue/stress groups, meaning that nurses with high fatigue/high stress profiles reported higher sleep-concern scores than those with low fatigue/low stress profiles. Sleep concerns also independently distinguished both higher-risk profiles from the low fatigue/low stress profile, consistent with the network analysis identifying the two sleep-concern subdimensions as bridging nodes.

Sleep concerns may represent candidate constructs for future longitudinal or intervention studies. Future studies could evaluate whether mindfulness-based stress reduction is associated with changes in sleep-related anxiety (60) and whether cognitive behavioral therapy for insomnia is associated with changes in fatigue or sleep-related cognitions among nurses (61).

### 4.4 Interrelationships among fatigue and stress within the network

Fatigue and stress were interconnected across several dimensions in the estimated network. One of the stronger positive associations was observed between sense of tension (CPSS1) and mental fatigue (MFI5). Persistent work stress has been associated with psychological tension and cognitive difficulties, including impaired concentration, memory problems, and slowed thinking (62). These cognitive difficulties may be related to work efficiency, coping with work challenges, and perceived stress; however, cognitive functioning was not directly assessed in the present study. Previous research has discussed sleep-dependent memory processing and brain-derived neurotrophic factor as possible explanations for cognitive impairment (63), but neither mechanism was examined in this study. Physical fatigue (MFI4) and sense of loss of control (CPSS2) occupied bridging positions linking fatigue and stress-related dimensions. Their association may be interpreted in relation to helplessness or resource depletion, but these processes were not directly measured, and the direction of association cannot be determined from the cross-sectional data (64). Physical fatigue and sense of loss of control may therefore represent candidate variables for future longitudinal or experimental investigation. Future studies could examine their temporal relationship and whether changes in one dimension are followed by changes in the other. Overall, these dimensions illustrate an interconnected pattern of fatigue and perceived stress rather than evidence of a causal or self-reinforcing cycle (65, 66).

LPA further identified heterogeneity in patterns of fatigue and perceived stress among nurses. These profiles may inform future evaluation of whether occupational-health support strategies should be tailored to nurses with different fatigue/stress patterns. Intervention research has suggested that structured support strategies, including mindfulness-based programs, may help reduce stress or burnout among nurses (67, 68). However, the present study does not establish that profile-based stratification improves intervention effectiveness. Future studies should therefore test whether nurses with different fatigue/stress profiles respond differently to support strategies of varying intensity and whether latent profiles can be used as a practical stratification tool in occupational-health planning.

The present findings are broadly consistent with international evidence. Studies among Jordanian nurses have linked sleep disturbance with fatigue and resilience with stress and quality of life (17, 51), while Australian qualitative research has described practical strategies used by nurses, midwives, and paramedics to manage sleep and fatigue during shift work (18). Other studies have linked nurses’ occupational stress with sleep quality and mental health and have associated shift-work characteristics with burnout (57, 65, 66). A broader theoretical review has synthesized occupational and organizational contributors to burnout in nursing (69). Evidence from the United States has linked extended working hours with patient-safety outcomes (70), while multinational research across Europe and the United States has associated nursing work environments with patient safety, satisfaction, and quality of care (71). The JD-R model provides a broader theoretical framework for interpreting these resource–strain and demand–strain associations (72). Nevertheless, replication of the present network structure and latent profiles in other countries and healthcare systems is required.

### 4.5 Practical implications

The findings suggest that future occupational-health research may benefit from evaluating integrated approaches that consider mental health promotion and sleep concerns together. A combination of network analysis and LPA revealed interconnected construct nodes and distinct fatigue/stress subgroups.

In resource-constrained clinical settings, the observed centrality of the values node may justify future investigation of professional meaning; however, professional meaning and any pathway linking it to fatigue or stress were not directly measured. Values (MHPS2) exhibited the strongest centrality and were negatively associated with physical fatigue. Therefore, professional values may be considered in future studies examining their associations with other psychological resources, fatigue, and stress. Further, APSQ2 (sleep-related preoccupation), which showed high bridge centrality values in the network, may be a candidate construct for future longitudinal or intervention research, including studies evaluating CBT-I, sleep hygiene education, or shift-system reforms without assuming effectiveness from the present data. These approaches warrant evaluation in relation to nurses’ long-term occupational health and career development, but their actual effectiveness requires validation in future longitudinal or intervention studies.

The identified fatigue/stress patterns may inform future evaluation of stratified support, including different levels of assistance for nurses with different profile characteristics. Accordingly, the observed central and bridge nodes should be used to generate hypotheses for future longitudinal or intervention studies, rather than to define immediate clinical priorities. Similarly, the statistically derived fatigue/stress profiles should be used as preliminary pattern descriptors for future validation, not as established clinical classifications. Therefore, future studies could evaluate the integration of subdimension-level and pattern-based assessments into routine occupational health management to help develop sustainable and comprehensive health promotion models, with the understanding that candidate constructs identified through network analysis are exploratory and are not established intervention targets.

### 4.6 Limitations and future directions

This study has several limitations. First, participants were recruited from three tertiary hospitals in one Chinese province, and 311 of the 338 participants (92.0%) were female. These sample characteristics may limit the generalizability of the findings to nurses in other regions, healthcare systems, levels of care, or more gender-balanced populations. The small male subgroup (*n* = 27) also limits the precision of gender-related estimates; therefore, associations involving gender should be interpreted cautiously and regarded as exploratory.

Second, all key variables were assessed using self-report scales at a single time point, creating risks of recall bias, social desirability bias, and common method bias. Shared method variance may have inflated associations among network nodes and the apparent centrality or bridge importance of some nodes, while consistent response tendencies may have increased the apparent separation of the latent profiles. In addition, using scale subdimension scores as network nodes may have obscured item-specific heterogeneity. Future studies should compare subdimension-level and item-level networks in larger samples and integrate the concurrently collected heart rate variability data or other objective indicators with self-report measures. Importantly, the HRV data were not analyzed in the present report; moreover, objective indicators measured at the same single time point as self-report scales would not, by themselves, establish temporal ordering or causality.

In addition, the low fatigue/low stress reference profile included only 33 participants, and some demographic categories were sparse. Although ridge regularization reduced coefficient instability and stratified bootstrap inference quantified uncertainty, some demographic estimates may remain imprecise and should be interpreted as exploratory. No model-specific *a priori* sample-size simulations were conducted for the network analysis or LPA, and the *post hoc* correlation-based sensitivity analysis did not directly assess conditional-edge recovery or latent-profile recovery. Accordingly, the network bootstrap and centrality-stability results and the LPA entropy, posterior probabilities, and class sizes support within-sample analytical performance but do not establish optimal sample adequacy or external replicability.

Finally, the study did not directly measure core occupational job demands, such as workload, emotional demands, staffing pressure, or shift intensity; therefore, the findings represent a partial JD-R-informed account rather than a comprehensive test of the JD-R model. Moreover, the cross-sectional design precludes conclusions about temporal ordering or causality among mental health promotion, sleep concerns, fatigue, and perceived stress. Future longitudinal, intensive longitudinal, ecological momentary assessment, and independent multicenter replication studies are needed to examine temporal dynamics and evaluate the robustness of the present findings.

## 5 Conclusion

The current study identified significant associations between mental health promotion and clinical nurses’ levels of sleep concerns, fatigue, and stress. Higher mental health promotion was associated with lower odds, and higher sleep concerns with higher odds, of membership in both the moderate fatigue/high stress and high fatigue/high stress profiles relative to the low fatigue/low stress profile. Among various psychological resources, values showed high centrality within the mental health promotion dimensions. Network analysis showed associations across multiple nodes linking mental health promotion and sleep concerns, thereby identifying candidate constructs for future longitudinal and intervention studies.

## Data Availability

The raw data supporting the conclusions of this article will be made available by the authors, without undue reservation.
