# Supplementary material for: Associations among mental health promotion, sleep concerns, fatigue, and stress in clinical nurses: network and latent profile analysis
Source: Front Public Health. 2026 Jul 16;14:1810766. doi: 10.3389/fpubh.2026.1810766 (PMC13422566; doi:10.3389/fpubh.2026.1810766)
Supplement: Supplementary file 1 [file Table_1.docx]

**Supplementary File 1**

**STROBE Checklist for Cross-Sectional Studies**

**Manuscript:** Associations among mental health promotion, sleep concerns, fatigue, and stress in clinical nurses: Network and latent profile analysis

Note: Locations below use section names so they remain valid after final pagination.

| **Section** | **Item** | **STROBE recommendation** | **Where reported** |
| --- | --- | --- | --- |
| Title and abstract | 1a | Identify the study design using a commonly used term in the title or abstract. | Abstract, Methods sentence (cross-sectional survey) |
| Title and abstract | 1b | Provide an informative and balanced summary of what was done and found. | Abstract |
| Introduction | 2 | Explain the scientific background and rationale. | Introduction, paragraphs 1–4 |
| Introduction | 3 | State specific objectives and any prespecified hypotheses. | Introduction, final paragraph (H1–H3) |
| Methods | 4 | Present key elements of the study design early in the paper. | Methods: Study design |
| Methods | 5 | Describe setting, locations, and relevant dates, including data-collection periods. | Methods: Study design and Data collection procedure (January–June 2025) |
| Methods | 6a | Give eligibility criteria and the sources and methods of participant selection. | Methods: Participants |
| Methods | 7 | Clearly define outcomes, exposures, predictors, confounders, and effect modifiers. | Methods: Measures; Statistical analyses |
| Methods | 8 | For each variable, give data sources and measurement methods. | Methods: Measures |
| Methods | 9 | Describe efforts to address potential sources of bias. | Methods: Data collection procedure (prespecified questionnaire screening); Limitations |
| Methods | 10 | Explain how the study size was determined. | Methods: Participants and Statistical analyses; Results: Participant flow and characteristics; Limitations and future directions |
| Methods | 11 | Explain how quantitative variables were handled and why any groupings were chosen. | Methods: Measures and Statistical analyses; Table 2 category definitions |
| Methods | 12a | Describe all statistical methods, including adjustment for confounding. | Methods: Statistical analyses (descriptive statistics, network analysis, latent profile analysis, profile comparisons, and ridge-penalized multinomial logistic regression) |
| Methods | 12b | Describe methods used to examine subgroups and interactions. | Methods: Statistical analyses (LPA and profile comparisons). No formal interaction analyses were performed. |
| Methods | 12c | Explain how missing data were addressed. | Methods: Participants and Statistical analyses (invalid questionnaires were excluded according to prespecified validity criteria; the final analytic dataset was checked for missing values; no imputation was required) |
| Methods | 12d | If applicable, describe analytical methods accounting for the sampling strategy. | Not applicable: no complex survey sampling |
| Methods | 12e | Describe any sensitivity analyses. | Methods and Results: post hoc correlation-based sensitivity analysis, bootstrap edge-weight accuracy analysis, and case-dropping centrality-stability analysis |
| Results | 13a | Report numbers at each stage of the study and reasons for non-participation or exclusion. | Results: Participant flow and characteristics (352 questionnaires returned; 14 excluded; 338 valid questionnaires analyzed) |
| Results | 13b | Give reasons for non-participation at each stage. | No non-participation occurred among the distributed questionnaires; reasons for exclusion of 14 returned questionnaires are reported in Results: Participant flow and characteristics. |
| Results | 13c | Consider use of a participant flow diagram. | Not used; participant flow is reported in text |
| Results | 14a | Give participant characteristics and information on exposures and potential confounders. | Results: Participant flow and characteristics; Table 2 |
| Results | 14b | Indicate the number of participants with missing data for each variable. | Methods: Statistical analyses; Results: Participant flow and characteristics (no missing data for any analyzed variable among the 338 participants) |
| Results | 14c | Summarize follow-up time, if relevant. | Not applicable to a cross-sectional study |
| Results | 15 | Report outcome data or summary measures. | Results; Tables 1–5; Figures 1–6 |
| Results | 16a | Give unadjusted and adjusted estimates with precision and identify adjusted confounders. | Tables 2–3 report univariate comparisons, and Tables 4–5 report multivariable ridge-adjusted estimates with bootstrap confidence intervals. |
| Results | 16b | Report category boundaries when continuous variables were categorized. | Table 2 and table notes |
| Results | 16c | If relevant, translate relative risk estimates into an absolute risk for a meaningful period. | Not applicable |
| Results | 17 | Report other analyses, including subgroup and sensitivity analyses. | Results: LPA profile comparisons and network stability analyses |
| Discussion | 18 | Summarize key results with reference to study objectives. | Discussion, opening paragraph |
| Discussion | 19 | Discuss limitations, including direction and magnitude of possible bias. | Limitations |
| Discussion | 20 | Provide a cautious overall interpretation considering objectives, limitations, multiplicity, and other evidence. | Discussion and Conclusion |
| Discussion | 21 | Discuss generalisability of the study results. | Limitations |
| Other information | 22 | State the source of funding and the role of funders. | Funding |

**Reporting guideline:** Strengthening the Reporting of Observational Studies in Epidemiology (STROBE), cross-sectional studies checklist.
